# What Denture Service Costs Dentists

**Published:** 1922-04

**Authors:** George Wood Clapp

**Affiliations:** New York


### WHAT DENTURE SERVICE COSTS DENTISTS

BY GEORGE WOOD CLAPP, D.D.S., NEW YORK

The purpose of this article is to present a brief analysis of what denture service appears to cost 38 dentists who report practices with gross annual receipts of less than $5,000. These practices show such individual differences that averages are of but slight value. The least profitable practice appears to be one with annual gross receipts of $2,000, and net receipts of $744. The most profitable practice appears to be one with annual gross receipts of $4,709, and net receipts of $3,744. Nothing is known about the accuracy of the accounting in either case.

Nothing is known about the number of income hours actually worked per year by the dentists who make these reports, and experience seems to show that in practices where there is not a good accounting system, the dentist is not likely to average much more than 1,000 income hours per year from the age of 35 to 55. The costs, as given in Column 6 of the table that follows, have been computed on the basis of 1,000 income hours per year. If the dentists are in the habit of working much more than that, the cost would be correspondingly reduced.

It is probable that the desired information can be better given by reproducing representative reports out of the group than by any report of averages.

In the following table Column 1 lists the reference number by which the practice is recorded. Column 2 records the annual gross receipts. Column 3 the annual net receipts. Column 4 shows the amount of chair time required by the dentist. Column 5 shows the amount of laboratory time. Column 6 shows the cost of the dentures to the dentist, including his remuneration, when based upon 1,000 income hours per year. Column 7 shows the habitual fee received by the dentist, if it is reported.

It should be borne in mind that in all these figures the work is identical; that is, the construction of full upper and lower vulcanite dentures for one mouth at one time.

| 1 | 2 | 3 | 4 | 5 | 6 | 7 |
|---|---|---|---|---|---|---|
| 86D | $1,580 | $1,210 | 8½ | 6½ | $23.70 | |
| 76D | 2,000 | 744 | 3 | 2½ | 11.00 | $50.00 |
| 27D | 2,560 | 1,290 | 6¼ | 14 | 51.84 | 60.00-80.00 |
| 89 | 3,241 | 2,616 | 4½ | 10 | 47.00 | 50.00 |
| 70D | 3,427 | 1,867 | 5½ | 4 | 33.58 | 50.00 |
| 83 | 3,700 | 2,800 | 4½ | 8 | 46.25 | |
| 42 | 3,854 | 1,949 | 6¼ | 30 | 139.56 | |
| 28 | 4,000 | 2,147 | 6 | 7 | 52.00 | |
| 46D | 4,024 | 2,224 | 4 | 2 | 29.12 | 50.00 |
| 40 | 4,200 | 2,400 | 6 | 7 | 54.60 | |
| 16D | 4,477 | 2,845 | 4¼ | 3½ | 34.15 | |
| 75 | 4,781 | 2,508 | 10 | 6 | 76.49 | |
| 46 | 4,954 | 3,258 | 10¾ | 30 | 201.71 | 100.00 |

One or more of three things is apparent: that the time records are often very imperfect or that some of the service is of a quality which ought to require the dentist to pay the patient or that there has been an extreme development of skill which permits the doing, especially of the laboratory work, in very short time.

Consider, for example, Practice No. 76D. The dentist requires three hours of chair time for the dentures. He does his own laboratory work but requires only two and a half hours of laboratory time to make full upper and lower vulcanite dentures. This includes making casts, attaching them to the articulator, making trial rims, arranging the teeth, waxing, flasking, vulcanizing and finishing. In Practice No. 46D, the dentist is even more rapid and does all the laboratory work upon the dentures in two hours.

Reports of cost based upon such time-reports are of course very misleading and should not be seriously considered by dentists who desire to render a quality of service satisfactory to themselves or to establish fees fair to both patient and dentist.

On the other hand, Practices No. 42 and No. 46 report rather more time than will probably be necessary, on the average, when these dentists are masters of the scientific technic they evidently follow. We have found, in the Research Division, that good service requires, on the average, about 30 hours for full upper and lower vulcanite dentures for one mouth at one time, but cases of such difficulty have occasionally presented as to require more than twice that length of time.

It is apparent that statements of costs and fees amount to very little unless related to a known quality of service, and that dentists who do not have good accounting systems may be basing their estimates of costs on false premises.

The variation in these reports and the very probable inaccuracies show plainly the importance of an exact accounting system in every practice, however small.—*Dental Digest.*

